# GRAM-CNN: a deep learning approach with local context for named entity recognition in biomedical text

**DOI:** 10.1093/bioinformatics/btx815

**Published:** 2017-12-20

**Authors:** Qile Zhu, Xiaolin Li, Ana Conesa, Cécile Pereira

**Affiliations:** 1 National Science Foundation Center for Big Learning, University of Florida, Gainesville, FL, USA; 2 Department of Computer & Information Science & Engineering, University of Florida, Gainesville, FL, USA; 3 Department of Electrical and Computer Engineering, University of Florida, Gainesville, FL, USA; 4 Department of Microbiology and Cell Science, Institute for Food and Agricultural Sciences, University of Florida, Gainesville, FL, USA; 5 Genomics of Gene Expression Laboratory, Centro de Investigación Príncipe Felipe, Valencia, Spain

## Abstract

**Motivation:**

Best performing named entity recognition (NER) methods for biomedical literature are based on hand-crafted features or task-specific rules, which are costly to produce and difficult to generalize to other corpora. End-to-end neural networks achieve state-of-the-art performance without hand-crafted features and task-specific knowledge in non-biomedical NER tasks. However, in the biomedical domain, using the same architecture does not yield competitive performance compared with conventional machine learning models.

**Results:**

We propose a novel end-to-end deep learning approach for biomedical NER tasks that leverages the local contexts based on n-gram character and word embeddings via Convolutional Neural Network (CNN). We call this approach GRAM-CNN. To automatically label a word, this method uses the local information around a word. Therefore, the GRAM-CNN method does not require any specific knowledge or feature engineering and can be theoretically applied to a wide range of existing NER problems. The GRAM-CNN approach was evaluated on three well-known biomedical datasets containing different BioNER entities. It obtained an F1-score of 87.26% on the Biocreative II dataset, 87.26% on the NCBI dataset and 72.57% on the JNLPBA dataset. Those results put GRAM-CNN in the lead of the biological NER methods. To the best of our knowledge, we are the first to apply CNN based structures to BioNER problems.

**Availability and implementation:**

The GRAM-CNN source code, datasets and pre-trained model are available online at: https://github.com/valdersoul/GRAM-CNN.

**Supplementary information:**

Supplementary data are available at *Bioinformatics* online.

## 1 Introduction

Named entity recognition (NER) is one of the first steps in the processing natural language texts. This task is aimed at identifying mentions of entities (e.g. persons, organizations and locations) in documents. In the biomedical domain, BioNER aims at automatically recognizing entities such as genes, proteins, diseases and species.

BioNER is considered more difficult than the general NER problem, because:

1. Millions of entities have been discovered, and the number is constantly increasing with the sequencing of new species.
2. The same biological entity can be described in different ways (Kim *et al.*, 2004).
3. The names of many biomedical entities are typically long (i.e. containing more than four words) (Zhou *et al.*, 2004).
4. Long sequences are usual in biomedical text.

There are several kinds of methods applied to extract named entities from biological texts. These include dictionary-based (Hirschman *et al.*, 2002), rule-based (Ananiadou, 1994), machine learning based (Collier *et al.*, 2000) and deep learning approaches (Limsopatham and Collier, 2016).

Dictionary-based approaches (Hirschman *et al.*, 2002) are limited by the size of the dictionary, misspellings, the use of synonyms and the constant increase of vocabulary. Rule-based approaches (Ananiadou, 1994; Tsai *et al.*, 2006), use common naming structures or morpho-syntactic features. These methods require extensive domain knowledge in order to develop rules, which are then not easily applicable to other domains. Machine learning-based methods suppose the initial definition of the features of interest. The most effective machine learning approaches applied to the NER problem are conditional random field approaches (CRF). The performance of CRF models rely heavily on the features, for example, orthographic, morphological, linguistic-based, conjunctions and dictionary-based. Those features are generally developed by experts, implying that they are task-specific and costly to develop. In this group of methods, we can cite ABNER (Settles, 2005), BANNER (Leaman *et al.*, 2008) and Gimli (Campos *et al.*, 2013).

Deep learning demonstrates state-of-the-art performance in many areas (LeCun *et al.*, 2015) including speech recognition (Hinton *et al.*, 2012), image classification (He *et al.*, 2016), image segmentation (Li *et al.*, 2016), part-of-speech (POS) tagging (Ma and Hovy, 2016) and NER (Lample *et al.*, 2016; Ma and Hovy, 2016). Fully connected neural network is used (Collobert *et al.*, 2011) to effectively identify entities in a newswire corpus. The application of character and word embeddings in Bi-directional Long Short-Term Memory (LSTM) (Lample *et al.*, 2016; Ma and Hovy, 2016) achieved state-of-the-art performance in several sequence-to-sequence datasets, such as CoNLL03 (Tjong Kim Sang and De Meulder, 2003) for NER and Penn Treebank WSJ (Marcus *et al.*, 1993) for POS tagging. Nevertheless, deep learning methods typically require a large amount of labeled data for supervised learning and take more time and computing resources to train than the classical machine learning methods.

Despite the good performance of the deep learning methods in many areas, the application of Bi-directional LSTM to the bioNER problem did not obtain as good results as conventional machine learning approaches (Limsopatham and Collier, 2016). Bi-directional LSTM uses the information contained in whole sentences. We hypothesized that long sentences could contain information unrelated with the target entities, and hence, in domains with long sentences, such as the biomedical literature, the utilization of local information rather than whole sentences may help improve precision.

Inspired in the inception model by (Szegedy *et al.*, 2015), we propose a novel neural network architecture to capture local information around each word in biomedical texts via Convolutional Nueral Network (CNN). To add some linguistic knowledge into our model, we also use POS tags as part of the input. This approach uses multiple n-gram features with different sizes together with its POS tag to capture each word’s environment. Our method, called GRAM-CNN, is an end-to-end model requiring no task-specific resources or handcrafted features. Details on CNN can be found in subsection 2.1.1.

The GRAM-CNN approach was evaluated on three biomedical datasets, Biocreative II (BC2) (Smith *et al.*, 2008), NCBI disease corpus (NCBI) (Doğan *et al.*, 2014) and the JNLPBA task (Kim *et al.*, 2004). It obtained an F1-score of 87.26% for BC2, 72.57% for JNLPBA and 87.26% for NCBI, always ranking among the top 2 best-performing methods. These results reveal that local information can efficiently predict the label of words and demonstrate that GRAM-CNN is a versatile approach that can theoretically be applied to wide range of BioNER tasks.

## 2 Materials and methods

Consider the following sentence from the BC2 dataset: ‘*STUDY DESIGN: Salivary immunoglobulin A levels of each of 20 subjects were determined on 3 occasions: first, while the subject was still smoking; second, 7 days after cessation of smoking; third, on the 14th day after cessation*’. In this sentence, the information about the study design (number of subjects and time points) is not relevant to understand that *Salivary Immunoglobulin A* is a protein. However, the words ‘*levels of each of 20 subjects*’, surrounding ‘*Salivary Immunoglobulin A*’, contain the term *level* that can successfully be used to tag *Salivary Immunoglobulin A* as a protein. Our approach focusses on this local context to better extract relevant information for the classification problem. We apply several convolutional kernel sizes (i.e. number of words around the target entity) to focus on the local context at multiple scales.

### 2.1 The GRAM-CNN method

**Fig. 1.**
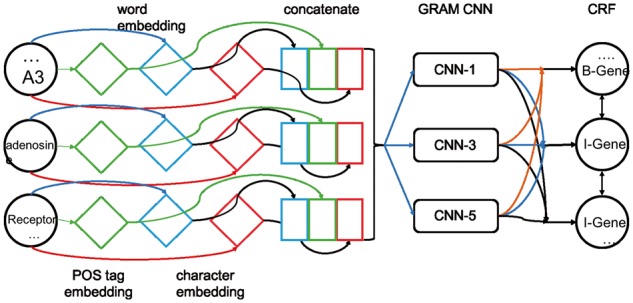
Overall scheme of the GRAM-CNN approach. First, we apply the POS tag (diamonds on the left), word (diamonds in the middle) and character (diamonds on the right) embedding and concatenate all embeddings into a combined vector (the 3 squares). Second, we feed the concatenated vector into the GRAM-CNN to retrieve the local context information. Finally, we model the predicted labels using CRF to get the final result. In this example, we use 3 different kernels in GRAM-CNN (1, 3, 5). The ‘B-Gene’, ‘I-Gene’ and ‘I-Gene’ are labels corresponding to ‘A3’, ‘adenosine’ and ‘Receptor’, respectively, in IOB tagging scheme (Sang and Veenstra, 1999). Further details of CNN-3 are given in Figure 3

The main steps of the GRAM-CNN method are as follows (Fig. 1):

1. Generate the word, POS tag and character embeddings.
2. Concatenate the character embeddings of each letter of a word with its word embedding and POS tag embedding.
3. Extract each word’s local features by GRAM-CNN with several kernel sizes as the final representation of each word (vector of CNN features) (see Fig. 3 for an example of kernel size 3).
4. Apply CRF to model labels jointly based on the output of GRAM-CNN.

In this section, the GRAM-CNN architecture is described in detail following the order from inputs to outputs, layer by layer.

#### 2.1.1 Embedding

Word embeddings bridge the gap between deep learning and natural language processing. Through this method, words are represented as dense vectors of real numbers, and those words that are semantically related are close in the high dimension space.

We used a pre-trained word embedding from biomedical texts proposed in 2016 (Chiu *et al.*, 2016) that recovered 87.34% of the words of the BC2 dataset. For the POS tag embedding, randomly initialized vectors were used to represent each tag. POS tags are extracted by the NLTK toolkit (Bird *et al.*, 2009). During training, we fixed the word embeddings and trained POS tag embedding together with the whole system.

**Fig. 2.**
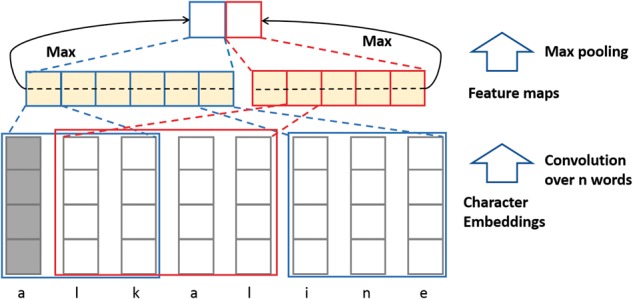
Diagram of the character embedding architecture. The vector of the letter *a* is in gray. In this example, CNN is applied with two different kernel sizes: 3 (a,l,k) and 4 (l, k, a,l), resulting in the production of two feature maps (middle squares). Then, a max pooling is applied to each feature map to get two representations of the word (top two squares in the figure). The length of embedding is two because in this example there is only one filter of each kernel size and two kernel sizes in total

The word embedding method cannot give a useful representation of words that are absent from the training vocabulary (out-of-vocabulary problem). To solve this issue, character level embedding from words was applied (Kim *et al.*, 2015). The character embedding was implemented with a CNN (Fig. 2).

In this implementation, each character in a word was represented by a vector of a fixed length *d*. A word with length *l* can be represented by a matrix *M *=* R^l^*^×^^*d*^. Each kernel *w* in CNN is a filter with shape *R^k^*^×^^*d*^, where k is the kernel size. The kernel size is equal to the size of a convolutional window across *k* characters.

(1) $$O_{i}=f(Conv(W,M_{t:t+k-1})+b).$$

*f* is the non-linear activation function (*tanh* in our experiments) and b is a bias vector. *Conv* is the convolution operation. *O* is the output vector of one kernel convolution with length l – *k* + 1. Its max value is used to represent one kernel’s feature. We used *k* = 2, 3, 4 and 40 filters for each *k*. Character embeddings were initialized randomly and trained with the whole network.

#### 2.1.2 GRAM-CNN

**Fig. 3.**
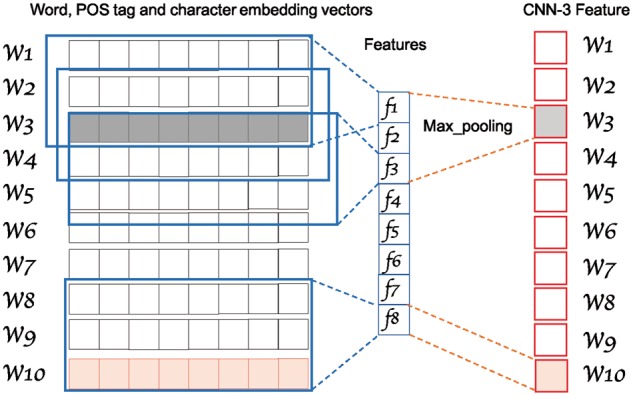
GRAM-CNN architecture. An example of kernel size 3 (squared) with an input of 10 words (word and character embedding concatenated) (on the left). CNN is used to extract information from these vectors. After the CNN step, a feature map is obtained (length 10 − 3 + 1 = 8 in this example). To extract the local information for each word, we use the correlated feature maps obtained partially with each word. A max pooling is applied over these feature maps, resulting in a vector of size one for each word (on the right). For example, the feature maps correlated with the word 3 (W3, in gray) are f1, f2, and f3 and the feature map correlated with W10 is f8

GRAM-CNN is a CNN model allowing to extract local information between a target word and its neighbors (Fig. 3). The representation of an input word is a vector concatenating pre-trained word embedding and character embedding. In GRAM-CNN, all convolutional filters process the same input at the same time, and this allows the model to take advantage of multi-level feature extractions with different kernel sizes (Szegedy *et al.*, 2015). This architecture is similar to character embedding mentioned in Section 2.1.1, and the only difference is the process of feature selection. GRAM-CNN is a sequence-to-sequence network. Each output of GRAM-CNN corresponds to one input. To achieve this, only the correlated features are selected of the word, i.e. features directly computed by this word and its neighbors. In this way, we were able to reduce the noise and get a better representation of the word.

To extract local information and n-gram features without breaking the internal relation between embeddings, we followed (Collobert *et al.*, 2011) using a filter with shape $R^{k\times (w_{e}+c_{e})}$ (*k* is the kernel size, *w_e_* is the length of word embedding and *c_e_* is the length of character embedding). Suppose we are selecting a word indexed by i in a sentence from feature maps *f_j_* convoluted by kernel size *j*.

(2) $$O_{i}=\text{tan}\;h(f_{j}[i-j+1:i])$$

A max pooling is operated after getting the outputs from same kernel size *j*. The outputs of the same word are concatenated into one vector. This vector is the representation of the word. In this work, 50 filters were used for each kernel size in GRAM-CNN.

Our goal is to predict a label for every word in the sentence. GRAM-CNN returns representations of each word. We then apply a two-layer fully connected network to get the final scores for each label of the word. These scores are sent to CRF to model the joint probability of words which will be described in the next subsection.

#### 2.1.3 CRF

A simple way to label each word is to use its own features to predict the label independently. This is a fast and effective strategy when labels are not correlated. However, entity names usually consist of several words, meaning that labels do have correlations with their neighborhoods. For example, in the IOB2 annotation (Sang and Veenstra, 1999), I-protein cannot follow B-gene or an O (outside of the entity) label. Therefore, it is beneficial to model the labels jointly. For this, a linear-chain conditional random field (CRF) (Lafferty *et al.*, 2001) is used.

For an input of a sentence containing n words, let *x_i_* denote the input vector of the *i_th_* word in the sentence, get $x=\left\{x_{1},\ldots ,x_{n}\right\}$. $y=\left\{y_{1},\ldots ,y_{n}\right\}$ is the potential sequence labels of *x*. *Y*(*x*) is the set of all possible label sequences. We used a variant of CRF, which is factored into unary potentials for single labels and binary potentials for every transition between output labels. The score was defined to be:

(3) $$s(x,y)=\sum_{i=0}^{n}T_{y_{i},y_{i+1}}+\sum_{i=1}^{n}P_{i,y_{i}}$$

The first part of the score function is the binary score while the second is a unitary score. $T_{y_{i},y_{i+1}}$ is the matrix of transition scores in which *T_i_*_,__*j*_ represents the score from tag *i* to *j*. *y*_0_ and *y_n_* are start and end tags in the sequence that are not part of the output sequence. *P* is a matrix with dimensions *n *×* t*, where n is the number of words in the sentence and t is the number of potential tags. $P_{i,y_{i}}$ corresponds to the score of *i*th word mapping to a specific tag, which is obtained from our neural network. The softmax function was used to compute the probabilities of all possible sequences.

(4) $$p(y|x)=\frac{e^{s(x,y)}}{\sum_{\bar{y}\in Y(x)}e^{s(x,\bar{y})}}$$

To train the CRF model, the log-likelihood of the probability was maximized. To predict the sequence label, the sequence that has the largest probability was selected:

(5) $$y^{*}=\underset{{}_{\bar{y}inY(x)}}{\text{argmax}}s(x,\bar{y})$$

Since the model binary potentials were the only modeled models, the Viterbi algorithm can solve the optimization problem and get an optimized result efficiently.

### 2.2 Implementation details

This section introduces how the network and parameters were trained. The GRAM-CNN approach, including the CRF layer was implemented by TensorFlow-v1.0.0 (Abadi *et al.*, 2015) and trained with one Nvidia Titan X GPU. We use the tokenizer and POS tagger from NLTK toolkit (Bird *et al.*, 2009) to preprocess each passage. Except for the pre-trained word embedding, all weights including character embedding and POS tag embedding were trained together. For character embedding, we use kernel sizes 2, 3, 4 and 40 filters for each size. We found that kernel size ranging from 1 to 10 is best for BC2 and NCBI datasets, while sizes 1 to 12 worked better for the JNLPBA dataset.

#### 2.2.1 Parameters initialization

We used a pre-trained word embedding from Chiu *et al.* (2016). For an unknown word (absent of the word embedding), the word ‘UNK’ was used to represent it, which implies that all unknown words had the same word embedding.

Character embedding was adopted to distinguish the unknown words. In order to allow the neural network to use both word and character embedding instead of focusing on a part of it, dropout layer (Srivastava *et al.*, 2014) was applied on this concatenated vector before the vector was input to GRAM-CNN.

For character embedding, 25 dimensions were used to represent the character with a uniform sample from $\left[-\sqrt{\frac{3}{dim}},+\sqrt{\frac{3}{dim}}\right]$ (He *et al.*, 2015) where *dim* is the embedding dimension. We used 15 dimensions for POS tag embedding with the same initialization. Both character and POS tag embedding were trained with the whole network. Xavier initialization (Glorot and Bengio, 2010) was used for all the convolutional layers and fully connect layers. All bias vectors were initialized to 0.

The kernel size decides how much local information (how many words) we take into account. Each kernel size can be seen as taking n-gram features from words. Different ranges of kernel sizes were tested to do experiments and compare to each on different datasets.

#### 2.2.2 Optimization method

Parameters in the network were optimized by stochastic gradient descent (SGD) with momentum 0.9. An initial learning rate of 0.002 with a learning decay = 0.95 was used. The learning rate was updated every 50 000 steps followed by $lr=lr*decay^{\left(steps/decaysteps\right)}$. Other sophisticated optimization algorithms such as AdaDelta (Zeiler, 2012) and Adam (Kingma and Ba, 2014) were also tried. None of them meaningfully improved on simple SGD using the momentum settings specified above.

#### 2.2.3 Tagging scheme

NER task is to assign every word in a sentence a label. A single entity may contain multiple words. IOB2 (Inside, Outside, Beginning) (Sang and Veenstra, 1999) tagging scheme was used to tag every word in the sentence. After tokenization, every token which is a start token of a named entity is labeled as a B-label. An I-label is assigned to a token if it is inside a named entity. Other words that do not belong to any named entities are labeled as an O-label. The word ‘label’ was replaced with the type of the named entity, for example, B-gene is a beginning token for a gene entity and I-gene is inside a gene entity.

### 2.3 Datasets

To evaluate our algorithm, three biomedical NER datasets were chosen: the BioCreative II Gene Mention task (BC2) (Smith *et al.*, 2008), the NCBI disease corpus (NCBI) (Doğan *et al.*, 2014) and the JNLPBA corpus (Kim *et al.*, 2004).

BC2 is concerned with the named entity extraction of gene and gene product mentioned in the text. The training and the test data are independent and composed of 15 000 and 5000 sentences, respectively. Since BC2 does not provide a development dataset, we created it with a ratio 3:1.

NCBI consists of 6892 disease mentions from 793 abstracts. Among those, 5145 are part of the training set, 787 are part of the development set, and 960 are part of the test set.

The JNLPBA dataset is a multi-entity dataset. It has protein, DNA, RNA, cell type and cell line, totaling five classes and representing a challenging scenario. It presents a training set and a test set of respectively 20 546 and 4260 sentences (51 301 and 8662 biomedical tags). The training set was divided with a ratio 3:1 in order to create the development set.

Training, validation and testing sets present in the NCBI dataset were used to evaluate these data. As the BC2 and JNLPBA datasets do not provide a validation set, their training datasets were split at a ratio 3:1 to created training and validation sets. The GRAM-CNN method was compared to the others NER methods already published and tested on the same data (Table 1).

**Table 1.** Datasets used to evaluate our approach. BC2 and JNLPBA do not provide separation of training and development datasets

	BC2	NCBI	JNLPBA
Target entities	Genes	Diseases	Protein, DNA, RNA, cell type and cell line
Type of data	sentences	mentions	sentences
Size of training set	10 000	5145	15 410
Size of development set	5000	787	5136
Size of test set	5000	960	4260

*Note*: We created them with a ratio 3:1.

### 2.4 Evaluation metrics

To evaluate the performance of the GRAM-CNN method and compare the results to other existing solutions, we used Precision, Recall and F-measure as experiment metrics:

(6) $$F1-score=\frac{2\cdot precision\;\cdot \;recall}{precision+recall}$$

(7) $$precision=\frac{TP}{TP+FP}$$

(8) $$recall=\frac{TP}{TP+FN}$$

Here TP (True Positive) is the number of entities that are correctly identified. FP (False Positive) is the number of chunks that are mistakenly identified as an entity. FN (False Negative) is the number of entities that are not identified. In BC2 and JNLPBA, the scripts provided along with the datasets were used to evaluate the performance. Precision represents the ability of a system to predict only true items, and recall makes sure that a system can predict all true items.

## 3 Results

The described GRAM-CNN method was applied to three different datasets and six different entities. Results were compared with other deep learning methods and conventional machine learning approaches.

### 3.1 BC2

Compared to other deep learning methods (Table 2), GRAM-CNN increased the previous best F1-score by 6.68%. With an F1-score of 87.26%, it was also the best method among the non-ensemble methods and ranked second on the BC2 dataset. GRAM-CNN performed better than the widely used BANNER (Leaman *et al.*, 2008), ABNER (Settles, 2005), Gimli (Campos *et al.*, 2013) and IBM (Ando, 2007). Moreover, among the top four methods, GRAM-CNN is the only one that does not require additional work. The IBM approach uses semi-supervised learning with additional data; AIIAGMT (Hsu *et al.*, 2008) ensembles eight different CRF models with two different CRF frameworks; and Gimli is an ensemble of several models. The GRAM-CNN neural network approach, end-to-end without ensemble and gazetteers, obtained a result of high quality without any additional data.

**Table 2.** Results of BC2 and NCBI datasets

Type	Ensemble	Approach	BC2				NCBI			
			Precision	Recall	F1	Rank	Precision	Recall	F1	Rank
M	N	ABNER (Settles, 2005)	86.93	54.49	64.68	11	−	−	−	−
D	N	FeedForward *	76.43	58.28	66.13	10	72.05	75.12	73.55	7
D	N	BiLSTM *	74.25	65.39	69.54	9	77.53	73.33	75.37	6
M	N	DNorm (Leaman *et al.*, 2013)	−	−	−	−	82.8	81.9	80.9	5
M	N	TaggerOne (Leaman and Lu, 2016)	−	−	−	−	85.1	80.8	82.9	4
M	N	NERBio (Tsai *et al.*, 2006)	**92.67**	68.91	79.05	8	−	−	−	−
D	N	CNN-BiLSTM *	80.75	79.76	80.25	7	84.33	84.06	84.17	3
D	N	ORTH-CNN-BiLSTM *	83.01	78.28	80.58	6	**86.67**	81.98	84.26	2
M	N	BANNER (Leaman *et al.*, 2008)	88.66	84.32	86.43	5	−	−	−	−
M	Y	Gimli (Campos *et al.*, 2013)	90.22	84.82	87.17	4	−	−	−	−
M	N	IBM (Ando, 2007)	88.48	85.97	87.21	3	−	−	−	−
D	N	GRAM-CNN (our method)	90.41	84.32	87.26	2	86.46	**88.07**	**87.26**	1
M	Y	AIIAGMT (Hsu *et al.*, 2008)	88.95	**87.65**	**88.30**	1	−	−	−	−

*Note*: * in the name of the approaches indicates result from (Limsopatham and Collier, 2016). D is short for ‘Deep learning’, M for ‘machine learning’. Y and N are ‘Yes’ and ‘No’. Best performance in each column is in bold.

**Table 3.** Results of JNLPBA dataset measured in F1 score

Approach	JNLPBA					
	Protein	DNA	RNA	Cell Type	Cell Line	Overall
POSBioTM (Song *et al.*, 2004)	69.07	60.08	64.07	64.**48**	57.33	66.28
Fin04 (Finkel *et al.*, 2004)	72.67	67.86	68.83	69.06	52.40	70.06
ABNER (Settles, 2005)	72.60	65.10	61.60	72.00	56.00	70.50
NERSuite (Tsai *et al.*, 2006)	72.74	68.58	67.23	72.11	56.11	71.07
GENIA Tagger (Tsuruoka *et al.*, 2005)	72.79	66.20	64.29	74.31	57.81	71.37
Gimli (Campos *et al.*, 2013)	74.68	69.27	67.24	70.49	58.64	72.23
Zho04 (Zhou *et al.*, 2004)	73.77	69.83	64.10	**75.13**	59.23	72.55
GRAM-CNN (our method)	74.37	68.64	66.95	73.58	**59.44**	72.57
NERBio (Tsai *et al.*, 2006)	**75.12**	**70.00**	**72.65**	72.77	57.39	**72.98**

*Note*: This corpus requires multi-class recognition (Protein, DNA, RNA, Cell Type, Cell Line), we compare each subclass’ score and overall score. The best score is in bold.

### 3.2 NCBI

The results obtained from the NCBI dataset are summarized in Table 2. DNorm (Leaman *et al.*, 2013) uses supervised semantic indexing, trained with pairwise learning to rank, then uses a CRF to return the score. TaggerOne (Leaman and Lu, 2016) was proposed recently, as it simultaneously performs NER and normalization and achieves an F1-score 82.9%. Deep learning methods play a leading role in the NCBI dataset. RNN with orthographic features has a result of 84.26% F1-score (Limsopatham and Collier, 2016).

Our method, outperforming all other tested algorithms in recall (88.07%) and F1-score (87.26%), ranked first on this dataset.

### 3.3 JNLPBA

JNLPBA corpus (Kim *et al.*, 2004) has protein, DNA, RNA, cell type and cell line totaling five different classes of entities. On this corpus, the top 3 methods presented an F1 score varying by less than 0.5% (Table 3).

NERBio (Tsai *et al.*, 2006), the best system on JLPBA corpus, obtained an F1-score of 72.98%. NERBio was implemented as a rule-based, post-processing approach that was designed especially for the JNLPBA task. On the BC2 dataset, NERBio only got an F1-score of 79.05%, which was under average and indicated that this solution is limited on other corpora.

In another hand, GRAM-CNN achieved an F1-score 72.57%. It outperformed most conventional machine learning systems and ranks second in the table with similar performance compared to NERBio. However, and contrary to NERBio, GRAM-CNN showed a high-performance on all the 3 tested datasets.

### 3.4 Error analysis

While GRAM-CNN was the one method that showed a constant top performance across different datasets, it was not free of mis-classifications. This section provides an error analysis of our method. We focused on the JNLPBA dataset, as this is a multi-class classification problem and therefore, more challenging than the NCBI and BC2 datasets.

Table 4 shows several types of errors obtained with the GRAM-CNN method on the JNLPBA test set.

**Table 4.** Examples of errors obtained with our approach applied on the JNLPBA test set

**Sentence example A**				
To investigate whether the tumor expression of beta2microglobulin *beta*2*M* could serve as a marker of tumor biologic behavior, the authors studied specimens of breast carcinomas from 60 consecutive female patients.				
**Ground Truth:**		**Detect:**		**Error type**
tumor	Cell_type			False negative
breast carcinomas	Cell_type			False negative
**Sentence example B**				
[1, 25-Dihydroxyvitamin D3 receptors in lymphocytes and T- and B-lymphocyte count in patients with glomerulonephritis].				
1, 25-Dihydroxyvitamin D3 receptors	Protein	[1, 25-Dihydroxyvitamin D3 receptors	Protein	Beginning
T- and B-lymphocyte	Cell_type	T-	Cell_type	Entity cut
		B-lymphocyte	Cell_type	
**Sentence example C**				
Analysis of the region 3’ to the CD4+ Tcell gene Rpt1 (encoding regulatory protein Tlymphocyte 1) led to the definition of a silencer element that inhibits heterologous gene expression in certain CD4+ Tcell lines but not in Bcell or nonlymphoid cell lines.				
CD4+ T-cell gene Rpt-1	DNA	CD4+ T-cell gene	DNA	Entity cut
		Rpt-1	Protein	
T-lymphocyte 1	Protein	regulatory protein T-lymphocyte 1	Protein	Entity include in a longer sentence
heterologous gene	DNA			False negative
B-cell or	Cell line	B-cell or non-lymphoid cell lines	Cell line	Consecutive entities regrouped
non-lymphoid cell lines	Cell line			
**Sentence example D**				
In the patients concentration of total and ionized form of Ca2+ was decreased down to 2.04 mmole/L and 1.09mmole/L, respectively, while an increase in parathormone (PTH) by 36% and a distinct decrease in 25 (OH) D concentration (lower than 1.25ng/ml) was found in blood content of cAMP was also decreased in lymphocytes by 33%.				
		lymphocytes	Cell_type	Error in the test set

In sentence A (Table 4), the word ‘tumor’ is described as a ‘Cell_type’ in the test set and was not predicted as a ‘Cell_type’ by GRAM_CNN. We noticed that, in the test set, the word tumor is associated with a cell type label in only 67% of the cases. This shows the difficulty for biocurators to be consistent in the annotation for nontrivial cases. This inconsistency was reflected in the predictions made by the NER methods.

Sentence B (Table 4) shows an example of misannotation of the border of the entity. In this case, the entity was recovered but the parenthesis situated just before the entity was seen as a part of the entity. A post-treatment of the protein name to ensure complete parenthesis-pairs could help solve this issue.

In sentence B, the GRAM-CNN method divided the reference to ‘T- and B-lymphocyte’ as two different entities: ‘T-’ and ‘B-lymphocyte’. In sentence C, a similar reference to entities ‘B-cell or non-lymphoid cell lines’ was considered this time as two different entities by the GRAM-CNN approach. Those differences in the annotation in the test set show a case where the annotation as one or two entities can both be considered as true and was treated inconsistently by the biocurators during their preparation of the test set.

Sentence C shows a case where an entity composed of several words ‘CD4+ T-cell gene Rpt-1’ was partially recovered and misannotated. In this case, GRAM-CNN cut the entities in two parts and labeled the first part ‘CD4+ T-cell gene’ as DNA but labeled the second part ‘Rpt-1’ as protein.

Sentence D presents an interesting case of false positive; ‘lymphocytes’ was recovered as a ‘Cell_type’ by GRAM-CNN but is not labeled in the test set. Since ‘lymphocytes’ is a cell type, this example is not a ‘real’ false positive of GRAM-CNN but a false negative case in the test set.

Among the randomly picked examples of errors of the GRAM-CNN method on the JNLPBA set, a fair proportion of errors corresponds to inconsistencies and errors in the test set. This could partially explain the smaller F1-score generally obtained by the NER methods on this test set compared to the results obtained from NCBI and BC2.

## 4 Discussion

The basic strategy of the GRAM-CNN is to address the BioNER problem by focussing on local information around each word rather than considering whole sequences as LSTM does. Several design choices contribute to the success of the GRAM-CNN method. First, GRAM-CNN uses a combination of word, character and POS tag embedding. The word embedding is pre-trained on biomedical text. The character embedding should provide the ability to represent new or misspelled words that are absent from the word embedding. Part-of-speech tag embedding provides the ability to take into account the grammatical information. Second, sentences in the biomedical text are typically longer than in other sources (14.53 words per sentence for CONLL-2013, 26.49 for JNKPBA). Several topics may be discussed in the same sentence and we hypothesize that local patterns can be found within these long sentences. In GRAM-CNN, the combination of multi-size CNN kernels focusing on the direct surrounding of each word reveals these local patterns, resulting in better performance than methods that merely considers the complete sentence, as is the case of LSTM.

Despite the fact that the GRAM-CNN method was successfully applied to three different datasets, some particular data structures are not yet supported. Our method is suitable for mentions consisting of a word or a group of consecutive set of words, and it’s also robust to misspellings. The three evaluated datasets fall into these categories. However, GRAM-CNN is not prepared to consider overlapping or disjoint mentions, neither mentions present in tables. Modifications to the IOB2 annotation (Sang and Veenstra, 1999) would be required to allow these possibilities. Supplementary Table S1 describes the type of mentions supported and not supported by GRAM-CNN.

As in all deep-learning methods, GRAM-CNN requires a significant amount of training data and is time-consuming. All the three tested datasets contain more than 5000 examples in the training set. A decrease of the quality of the assignation is expected if GRAM-CNN is trained on a smaller dataset. Training time is longer compared with conventional machine learning methods. The network converged after about 100 epochs for all three datasets. For JNLPBA and BC2 datasets, it takes about 5 and 1.5 days for NCBI dataset. However, once training is finished, inference on a test set is comparatively fast, taking from 2 to 5 min to complete on around 5000 sentences.

Finally, it was recently shown that combining NER and normalization can improve the performance (Leaman and Lu, 2016). This suggests that within a multi-task architecture, combining our GRAM-CNN approach with a normalization procedure may improve performance. We anticipate that entities containing conjunctions and punctuations usual in biomedical NER will remain difficult to handle after normalization, which will still be a challenge for our and other approaches.

## 5 Conclusion

We hypothesized that local context information plays an important role in biomedical NER tasks. We implemented GRAM-CNN, a novel end-to-end neural network using both character embedding and word embedding for the biomedical NER tasks. This method, without using any hand-crafted features or domain knowledge, ranked among the top 2 methods on each tested dataset and achieved F1-scores of 87.26% in the BC2 dataset, 87.26% in NCBI dataset and 72.57% in JNLPBA dataset. To the best of our knowledge, this method is the first to achieve competitive performance using deep learning compared with conventional machine learning approaches in biomedical NER.

By applying the GRAM-CNN method on three different datasets, we showed that the GRAM-CNN approach is a versatile approach that can be widely applied to BioNER problems without requiring any hand-crafted features or humanly designed rules.

## Funding

This material is based upon work that was supported in part by the National Institute of Food and Agriculture, U.S. Department of Agriculture http://nifa.usda.gov/ (Award number 2015-70016-23029), National Science Foundation (grants ACI 1245880, ACI 1229576, CCF-1128805, CNS-1624782) and National Institutes of Health (R01GM110240). The content is solely the responsibility of the authors and does not necessarily represent the official views of the granting agency.

*Conflict of Interest*: none declared.

## Supplementary Material

Supplementary Data
