# Supplementary material for: GRAM-CNN: a deep learning approach with local context for named entity recognition in biomedical text
Source: Bioinformatics. 2017 Dec 20;34(9):1547–54. doi: 10.1093/bioinformatics/btx815 (PMC5925775; doi:10.1093/bioinformatics/btx815)
Supplement: Supplementary Data [file btx815_gram-cnn_supplementary.pdf]

Supplementary

Type of mention	Example			Supported?
	Sentence	Ground Truth		
Mono-label	Rpt-1 is express	Rpt-1	Protein	YES
Multi-label	Analysis of the region 3' to the CD4+ T-cell gene Rpt-1 (encoding regulatory protein T-lymphocyte 1) led to the definition of a silencer element	CD4+ T-cell gene Rpt-1	DNA	YES
		T-lymphocyte 1	Protein	
Disjoin mentions	The left atrium is severely dilated	left atrium dilated	label A	NO
Misspell word	blood content of cAMP was also decreased in lymphcytes by 33%	lymphocytes	Cell_type	YES

Table 1: Example of mentions supported or not by the current implementation of the GRAM-CNN method. In the misspell word setting, words are treated as unknown words, it can be recovered by character-embedding. In this example, expected is 'lymphcytes'.
